# Genetic Insight into Disease Resistance Gene Clusters by Using Sequencing-Based Fine Mapping in Sunflower (*Helianthus annuus* L.)

**DOI:** 10.3390/ijms23179516

**Published:** 2022-08-23

**Authors:** Guojia Ma, Qijian Song, Xuehui Li, Lili Qi

**Affiliations:** 1 Department of Plant Sciences, North Dakota State University, Fargo, ND 58102-6050, USA; 2 Soybean Genomics and Improvement Laboratory, USDA-Agricultural Research Service, Beltsville, MD 20705-2350, USA; 3 USDA-Agricultural Research Service, Edward T. Schafer Agricultural Research Center, Fargo, ND 58102-2765, USA

**Keywords:** sunflower, rust, downy mildew, resistance genes, fine mapping

## Abstract

Rust and downy mildew (DM) are two important sunflower diseases that lead to significant yield losses globally. The use of resistant hybrids to control rust and DM in sunflower has a long history. The rust resistance genes, *R_13a_* and *R_16_*, were previously mapped to a 3.4 Mb region at the lower end of sunflower chromosome 13, while the DM resistance gene, *Pl_33_*, was previously mapped to a 4.2 Mb region located at the upper end of chromosome 4. High-resolution fine mapping was conducted using whole genome sequencing of HA-R6 (*R_13a_*) and TX16R (*R_16_* and *Pl_33_*) and large segregated populations. *R_13a_* and *R_16_* were fine mapped to a 0.48 cM region in chromosome 13 corresponding to a 790 kb physical interval on the XRQr1.0 genome assembly. Four disease defense-related genes with nucleotide-binding leucine-rich repeat (NLR) motifs were found in this region from XRQr1.0 gene annotation as candidate genes for *R_13a_* and *R_16_*. *Pl_33_* was fine mapped to a 0.04 cM region in chromosome 4 corresponding to a 63 kb physical interval. One NLR gene, HanXRQChr04g0095641, was predicted as the candidate gene for *Pl_33_*. The diagnostic SNP markers developed for each gene in the current study will facilitate marker-assisted selections of resistance genes in sunflower breeding programs.

## 1. Introduction

Sunflower (*Helianthus annuus* L.) is among the few crops that are native to North America [1,2,3]. Based on the use of its products, sunflower can be classified as confectionary sunflower for human consumption, oilseed sunflower for edible oil, and ornamental sunflower. In addition to these routine uses, environmental scientists have found that sunflower plants can absorb high concentrations of toxic chemicals from soil into their tissues leaves and stems. It has been demonstrated to be a success in industry when scientists use sunflower to clean up land contaminated with lead (https://gardencollage.com/change/sustainability/scientists-using-sunflowers-clean-nuclear-radiation/ (accessed on 10 July 2022)). Although sunflower can tolerate toxic environments and adapt to different agroecological conditions, its growth is still challenged by many biotic and abiotic stresses throughout its life cycle. Downy mildew (DM) and rust are two of the most devastating diseases that impair sunflower production worldwide.

Downy mildew, which is caused by the oomycete pathogen, *Plasmopara halstedii* (Farl.) Berl. & de Toni, is one of the most damaging diseases in sunflower globally. In epidemic years with cool and wet weather, yield loss can be as high as 95% [4]. As one of the most dynamic pathogens, a total of 44 *P. halstedii* races have been recorded worldwide, with more than 24 *P. halstedii* races reported in Europe and 40 in the Americas [5,6,7,8]. The use of resistant hybrids is the first choice for disease management in sunflower for economic and environmental reasons. Host race-specific resistance genes against DM, designated *Pl*, have been utilized on a commercial scale for sunflower production since the 1970s [9,10]. To date, a total of 37 *Pl* genes, *Pl_1_*–*Pl_36_*, and *Pl_Arg_*, have been identified and reported from a resistance gene (*R* gene) pool that encompasses both cultivated and wild sunflowers (Supplementary Table S1) [11,12,13,14,15,16,17,18,19,20,21,22,23,24,25,26,27,28,29,30,31,32,33,34,35,36,37,38,39,40,41,42,43,44]. Thirty-one of them have been located on different chromosomes across the sunflower genome: chromosome 1 (*Pl_Arg_*, *Pl_13_*, *Pl_14_*, *Pl_16_*, *Pl_23_*–*Pl_25_*, and *Pl_35_*); chromosome 2 (*Pl_18_* and *Pl_26_*); chromosome 4 (*Pl_17_*, *Pl_19_*, *Pl_27_*–*Pl_29_*, and *Pl_33_*); chromosome 8 (*Pl_1_*, *Pl_2_*, *Pl_6_*, *Pl_7_*, *Pl_15_*, and *Pl_20_*); chromosome 11 (*Pl_30_*); and chromosome 13 (*Pl_5_*, *Pl_8_*, *Pl_21_*, *Pl_22_*, *Pl_31_*, *Pl_32_*, *Pl_34_*, and *Pl_36_*).

Rust, which is caused by the fungus, *Puccinia helianthi* Schw., is another severe sunflower disease that is present around the world. After infection, sunflower plants can still grow; however, both the yields and seed quality will be reduced. Yield losses as high as 80% can occur in epidemic years [45]. A recent survey coordinated by the USA National Sunflower Association indicated that rust is the most prevalent disease among the common sunflower diseases that have been investigated [46]. A total of 39 *P. helianthi* races were identified in North America, in which races 334 and 336 were predominant, while race 777 was the most virulent [47]. Identification of *P. helianthi* races was also reported in Australia, Argentina, China, and South Africa [48,49,50,51]. Similar to that of DM, the use of sunflower host resistance is the top choice for rust management. Rust resistance in sunflower is controlled by single dominant genes. To date, a total of 17 rust resistance genes, *R_1_*–*R_5_*, *R_10_*–*R_12_*, *R_13a_*, *R_13b_*, *R_14_*–*R_18_*, *P_u6_* and *R_adv,_* have been reported in sunflower, and 15 of them were mapped to various regions across the sunflower genome: chromosome 2 (*R_5_*); chromosome 8 (*R_1_* and *R_15_*); chromosome 11 (*R_12_* and *R_14_*); chromosome 13 (*R_4_*, *R_11_*, *R_13a_*, *R_13b_*, *R_16_*–*R_18_*, *P_u6_*, and *R_adv_*); and chromosome 14 (*R_2_*) (Supplementary Table S2) [52,53,54,55,56,57,58,59,60,61,62,63,64,65,66,67,68,69,70,71,72,73,74].

The rust *R* genes, *R_13a_*, *R_13b_*, and *R_16_*, were previously mapped to a large gene cluster located at the lower end of chromosome 13 and delimited in a 3.4 Mb region in the XRQr1.0 genome assembly [40,64,75]. No *P. helianthi* race can differentiate among *R_13a_*, *R_13b_*, and *R_16_*, and no existing markers can be used to saturate the target gene region. High-resolution mapping assisted by whole genome sequencing is needed to further examine this region and to develop diagnostic markers for each of the *R* genes in the cluster. This would be essential for promptly and accurately incorporating new resistance genes into elite sunflower breeding lines through marker-assisted selection (MAS), as TX16R carrying a rust *R* gene *R_16_* and DM *R* gene *Pl_33_* is still resistant to all *P. halstedii* and *P. helianthi* races identified thus far after release in 2005 and has not been widely used in sunflower breeding [40].

The DM *R* gene *Pl_33_* in the TX16R line was initially mapped to the upper end of sunflower chromosome 4 and was co-segregated with two simple sequence repeat (SSR) markers and two single nucleotide polymorphism (SNP) markers [40]. Two additional DM *R* genes, *Pl_17_* and *Pl_19_*, were also found within this interval. The *Pl_17_* and *Pl_19_* markers can differentiate among the three *R* genes, indicating that the three genes were independent of each other [76]. However, further saturation of the *Pl_33_* interval in chromosome 4 is needed to facilitate specific gene introgressions. In this study, we report on the fine mapping of three *R* genes, *R_13a_*, *R_16_*, and *Pl_33_*, by using a sequencing-based marker development approach combined with high-density mapping populations. The diagnostic SNP markers that were developed in this study for each targeted gene will facilitate MAS and gene pyramiding in sunflower breeding programs. Our current study provides a foundation and new genetic resource for the cloning of these genes in the future.

## 2. Results

### 2.1. Saturation and Fine Mapping of R_13a_

**Figure 1:**
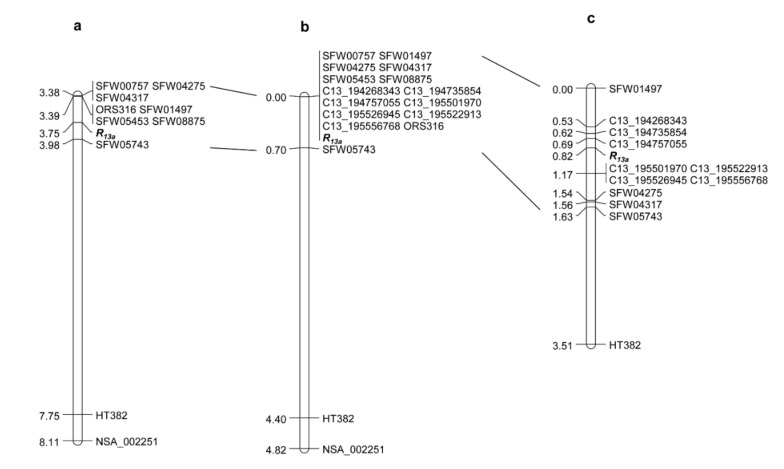
Rust *R_13a_* gene linkage maps. (**a**) Map taken from Qi et al., 2015 [75], (**b**) *R_13a_* saturation map, and (**c**) *R_13a_* fine map.

Previous genetic mapping from a population of the cross between rust susceptible-HA 89 and rust resistant-HA-R6 placed the rust *R* gene *R_13a_* derived from HA-R6 in a 0.59 cM region located at the lower end of chromosome 13. *R_13a_* was flanked by SNP marker SFW05743 and a group of co-segregated markers (Figure 1a) [75], which corresponded to a 3.4 Mb physical interval between 193.1–196.5 Mb in the XRQr1.0 genome assembly and a 1.8 Mb physical interval between 236.4–238.2 Mb in the HA412-HO genome assembly, respectively (Table 1). A total of 432 SNP markers were selected from whole-genome sequencing in the target region and were screened for polymorphisms between parents, HA 89 and HA-R6 (*R_13a_*). The identified polymorphic markers were further used to genotype the F_2_ population with 140 individuals, and seven SNPs were mapped to the *R_13a_* target region and all were selected from the XRQr1.0 assembly. Due to a small population size of 140 and small number of markers mapped to *R_13a_*, the saturation mapping step assigned the *R_13a_* gene to co-segregate with a cluster of 14 markers (Figure 1b).

To dissect the marker cluster and increase the map resolution of *R_13a_*, a large population consisting of the 2820 F_3_ individuals selected from the F_3_ families that were heterozygous for *R_13a_* was screened using two flanking markers, the SNP marker SFW01497 and the SSR marker HT382. A total of 312 F_3_ recombinants were identified in the target region that was delimited by these two markers and advanced to the next generation for rust testing of the recombinant families. Seven SNP markers in the saturation map were used to genotype the 312 recombinants identified from the large population. The combined phenotype and marker data of the recombinants placed *R_13a_* in a 0.48 cM interval flanked by SNP marker C13_194757055 (0.13 cM) and a cluster of four SNP markers, C13_195501970, C13_195522913, C13_195526945 and C13_195556768 (0.35 cM) (Figure 1c). This genetic region corresponds to a 745 kb segment in the XRQr1.0 assembly, which decreases the *R_13a_* physical interval from 3.4 Mb to 0.745 Mb (Table 1). The genetic positions of the mapped SNP markers of *R_13a_* agree well with their physical positions for both XRQr1.0 and HA412-HO, except for the physical position of C13_194735854 on the HA412-HO assembly (Table 1).

### 2.2. Saturation and Fine Mapping of R_16_

**Figure 2:**
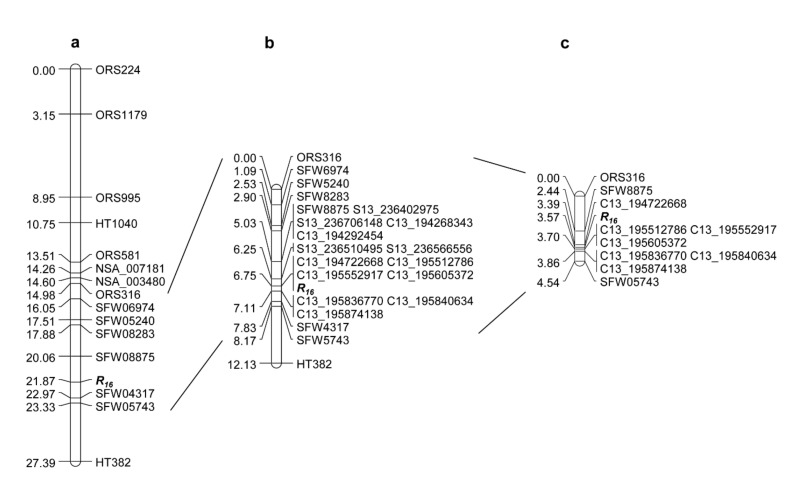
Rust *R_16_* gene linkage maps. (**a**) Map taken from Liu et al., 2019 [40], (**b**) *R_16_* saturation map, and (**c**) *R_16_* fine map.

Genetic mapping of *R_16_* was initially performed in a population derived from the cross between rust and DM susceptible-HA 434 line and rust and DM resistant-TX16R line [40]. The rust *R* gene *R_16_* from TXR16 was previously mapped into a 2.91 cM interval flanked by public SNP markers, SFW08875 and SFW04317, in a region with *R_13a_* and *R_13b_* on sunflower chromosome 13 (Figure 2a), which corresponded to 3.4 Mb and 2.0 Mb regions in the XRQr1.0 and HA412-HO assemblies, respectively [40]. A total of 432 SNP markers that were selected based on SNPs/InDels between HA-R6 (*R_13a_*)/TX16R (*R_16_*) and two reference genomes in the target region of chromosome 13 were first used to screen for polymorphisms between parents HA 434 and TX16R. Polymorphic markers were further selected to genotype the F_2_ population of HA 434 × TX16R with 146 individuals. Fifteen new SNP markers were mapped around *R_16_*, which delimited *R_16_* to a 0.86 cM interval (Figure 2b, Table 2). In the saturation map, *R_16_* co-segregated with four SNP markers, C13_194722668, C13_195512786, C13_195552917, and C13_195605372, and was flanked by two SNP clusters (Figure 2b).

To dissect marker clusters and further improve the map resolution of *R_16_*, an F_2:3_ population of 2256 individuals that were segregated for *R_16_* were screened with two flanking markers, the SSR marker ORS316 and the SNP marker SFW05743. A total of 203 F_3_ recombinants were identified in the target region that was defined by these two markers and advanced to the next generation for rust testing of the recombinant families. The 10 SNP markers selected from the saturation map were used to genotype the 203 recombinants. The marker data were further analyzed with rust phenotyping data, resulting in *R_16_* being narrowed down to a 0.31 cM interval, which corresponded to a 790 kb region in the XRQr1.0 assembly (Figure 2c, Table 2). After fine mapping, *R_16_* was flanked by SNP marker C13_194722668 (0.18 cM distance) and a cluster of three SNP markers, C13_195512786, C13_195552917 and C13_195605372 (0.13 cM distance) (Figure 2c). The mapped SNP markers for *R_16_* were physically in agreement with their genetic positions on the XRQr1.0 assembly (Table 2).

### 2.3. Saturation and Fine Mapping of Pl_33_

**Figure 3:**
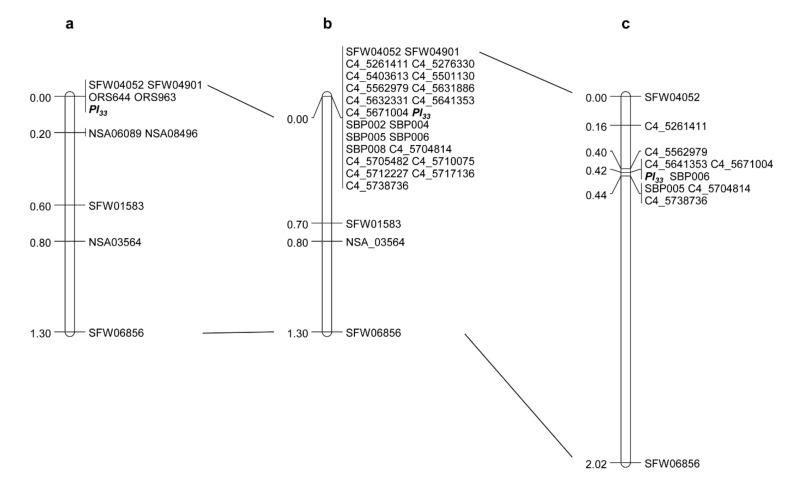
DM *Pl_33_* gene linkage maps. (**a**) Map taken from Liu et al., 2019 [40], (**b**) *Pl_33_* saturation map, and (**c**) *Pl_33_* fine map.

As the above population from the cross HA 434 and TX16R also segregated for DM resistance, this population was also used for genetic mapping of the DM *R* gene *Pl_33_* from TX16R. *Pl_33_* was previously mapped to a similar position as *Pl_17_* on sunflower chromosome 4 and was co-segregated with SSR markers, ORS644 and ORS963, and SNP markers, SFW04052 and SFW04901 (Figure 1a) [40]. In the present study, a total of 157 SNP markers selected in the target region from the variants between TX16R (*Pl_33_*)/HA 458 (*Pl_17_*) sequences and the XRQr1.0 sequence were first screened for polymorphisms between HA 434 and TX16R. Subsequently, the polymorphic markers were used to genotype the 148 F_2_ individuals in the HA 434 × TX16R population. A total of 23 SNPs, 15 from variants between HA 458 (*Pl_17_*) and the XRQr1.0 reference and 8 from variants between TX16R (*Pl_33_*) and the XRQr1.0 reference, were mapped around *Pl_33_*, which led to the co-segregation of *Pl_33_* with a cluster of 22 markers (Figure 3b).

To differentiate the co-segregated marker clusters, *Pl_33_* flanking SNP markers, SFW04052 and SFW06856, were selected to screen the same F_2:3_ population of 2256 individuals that were segregated for both *R_16_* and *Pl_33_* as used above. A total of 111 recombinants located in the target region were identified and advanced to the next generation for DM testing. The 10 mapped SNP markers selected from the saturation map were used to genotype the 111 *Pl_33_* recombinants to increase the *Pl_33_* map resolution. After linkage analysis using DM phenotyping data, the *Pl_33_* gene was placed in a 0.04 cM interval on chromosome 4, which corresponded to a 63 kb interval in the XRQr1.0 genome assembly (Figure 3c, Table 3). *Pl_33_* was co-segregated with three SNP markers, C4_5641353, C4_5671004, and SPB006, and was flanked by C4_5562979 (0.02 cM) and a cluster of three markers, SPB005, C4_5704814, and C4_5738736 (0.02 cM) (Figure 3c, Table 3). The genetic positions of the SNP markers in the fine map agree well with their physical positions on the XRQr1.0 assembly on chromosome 4 but do not agree well with their physical positions on the HA412-HO assembly, in which SNP marker C4_5641353 was mapped to a distant position located outside of the interval, and most of the whole genome sequence-based SNP markers from the XRQr1.0 genome had a reversed order in the HA412-HO assembly (Table 3).

### 2.4. Comparative Analysis of R_13a_, R_13b_, and R_16_ on Chromosome 13

**Figure 4:**
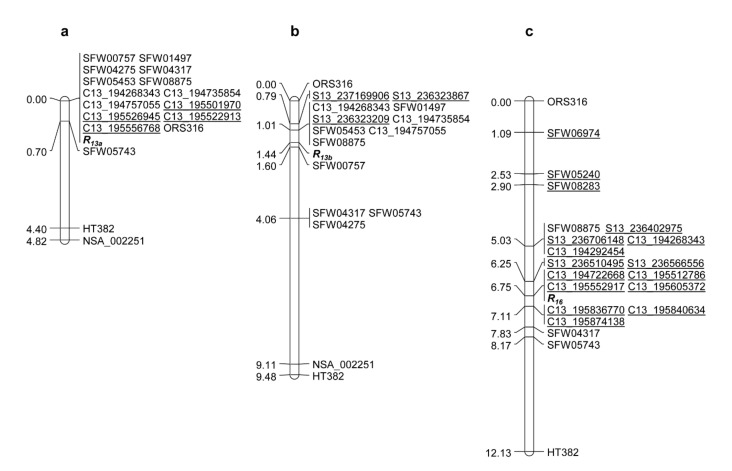
Genetic maps of *R_13a_*, *R_13b_* and *R_16_*. (**a**) *R_13a_* saturation map, (**b**) *R_13b_* saturation map, and (**c**) *R_16_* saturation map. The underlined markers are the unique markers in each map.

*R_13a_* and *R_13b_* were previously mapped together on sunflower chromosome 13 by using the same sets of SSR and SNP markers obtained from published genetic maps, and no marker could differentiate these two genes [64,75]. In the current study, the same set of 432 SNPs that was used in the *R_13a_* and *R_16_* saturation mapping was also used to screen polymorphisms between the parents, HA 89 and RHA 397 (*R_13b_*). Six SNPs were polymorphic and were subsequently used to genotype the 140 F_2_ individuals derived from the HA 89/RHA 397 cross. All six SNPs were mapped distal to *R_13b_* (Figure 4b). Comparative analysis of the *R_13a_* and *R_13b_* saturation maps revealed that four SNP markers, C13_195501970, C13_195522913, C13_195526945, and C13_195556768, could differentiate *R_13a_* from *R_13b_*, while three SNP markers, S13_236323209, S13_236323867, and S13_237169906, could differentiate *R_13b_* from *R_13a_*, which suggested that they are different genes (Figure 4a,b). Twelve SNP markers in the *R_16_* saturation map developed in the current study could differentiate *R_16_* from *R_13a_* and *R_13b_* (Figure 4c). The genetic positions of the mapped SNP markers from the saturation maps of *R_13a_*, *R_13b_*, and *R_16_* and the fine maps of *R_13a_* and *R_16_* are summarized in Table 4.

### 2.5. Comparative Analysis of Pl_17_, Pl_19_, and Pl_33_ on Chromosome 4

*Pl_17_*, *Pl_19_*, and *Pl_33_* are all located in an *R* gene cluster on sunflower chromosome 4 [32,34,40,76]. Genetic dissection by sequencing-based fine mapping revealed that *Pl_19_* is located 1 Mb from *Pl_17_* (Table 5) [76]. In the present study, in addition to the SNPs selected between TX16R (*Pl_33_*) and the XRQr1.0 reference, a set of 129 SNP markers used in *Pl_17_* fine mapping that was selected between HA 458 (*Pl_17_*) and the XRQr1.0 reference was also used for saturation and fine mapping of *Pl_33_*. These common markers shared between *Pl_17_* and *Pl_33_* can clearly distinguish the two genes, which indicates that *Pl_17_* is proximal to *Pl_33_* (Table 5). Although *Pl_17_* and *Pl_33_* are located close together in a small region between SNP markers C4_5671004 and SPB001 on chromosome 4, each gene has its own diagnostic markers, which facilitates introduction of each gene into elite sunflower lines and gene pyramiding in sunflower breeding programs (Table 5).

### 2.6. Candidate Gene Analysis of R_13a_, R_16_, and Pl_33_

In the current study, both *R_13a_* and *R_16_* were fine mapped to a 790.5 kb region between nucleotide positions of 194,722,468 and 195,512,986 bp on chromosome 13 of the XRQr1.0 genome assembly (Table 1 and Table 2). The four predicted plant disease defense-related genes were found in the target region, which encodes the putative NB-ARC domain, a signaling motif shared by plant resistance gene products (Table 6). In a 63.4 kb *Pl_33_* target region between nucleotide positions of 5,641,153 and 5,704,545 bp on chromosome 4 of the XRQr1.0 genome assembly (Table 3), only one gene, HanXRQChr04g0095641, was predicted to be a probable disease resistance protein (TIR-NBS-LRR class) family, which is the same candidate gene as *Pl_17_* (Table 6) [77].

### 2.7. Identification of Diagnostic Markers for R_13a_, R_16_, and Pl_33_

**Figure 5:**
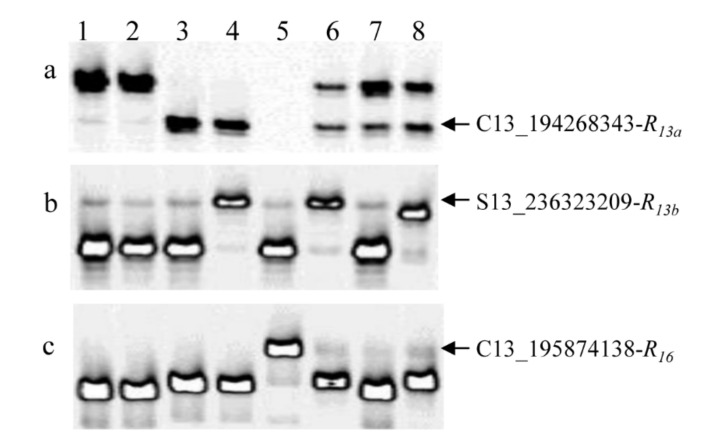
The polymerase chain reaction (PCR) amplification patterns of the single nucleotide polymorphism (SNP) markers in the eight sunflower lines. (**a**) SNP C13_194268343 linked to *R_13a_*, the arrow indicates the amplified band corresponding to C13_194268343-*R_13a_* marker allele, *R_13a_* shares the PCR pattern of the marker allele with *R_13b_*. (**b**) SNP S13_236323209 linked to *R_13b_*, the arrow indicates the amplified band corresponding to S13_236323209-*R_13b_* marker allele, *R_13b_* shares the PCR pattern of the marker allele with *R_4_*. (**c**) SNP C13_195874138 linked to *R_16_* can distinguish *R_16_* from all genes in the cluster, the arrow indicates the amplified band corresponding to C13_195874138-*R_16_* marker allele. Lane 1, HA 89; Lane 2, HA 434; Lane 3, HA-R6/*R_13a_*; Lane 4, RHA 397/*R_13b_*; Lane 5, TX16R/*R_16_*; Lane 6, HA-R3/*R_4_*; Lane 7, HA-R18/*R_17_*; Lane 8, HA-R19/*R_18_*.

**Figure 6:**
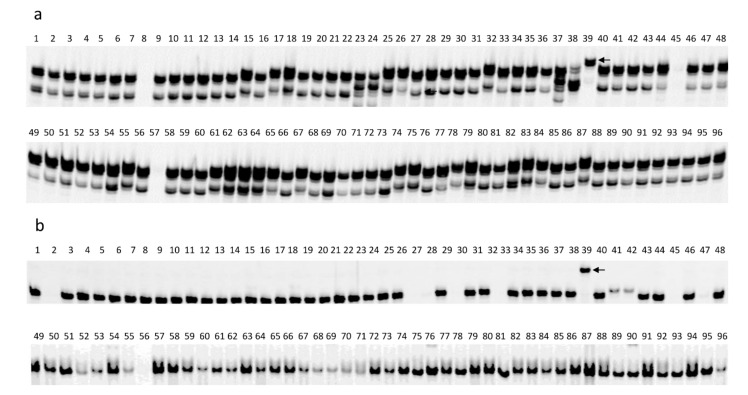
The polymerase chain reaction (PCR) amplification pattern of single nucleotide polymorphism (SNP) markers in the 96 selected sunflower lines. The names and pedigrees of 96 selected sunflower lines (lanes) are listed in Supplementary Table S3. (**a**) SNP marker C13_194722668 diagnostic for *R_16_*, the arrow indicates the amplified band corresponding to C13_194722668-*R_16_* marker allele which is only present in the TX16R line in lane 39. (**b**) SNP marker C4_5671004 diagnostic for *Pl_33_*, the arrow indicates the amplified band corresponding to C4_5671004-*Pl_33_* marker allele which is only present in the TX16R line in lane 39. Lane 39: TX16R with *R_16_* and *Pl_33_*.

Currently, six rust *R* genes, *R_4_*, *R_13a_*, *R_13b_*, *R_16_*, *R_17_*, and *R_18_*, are located in a similar region on sunflower chromosome 13 [40,57,64,73,75]. A total of 16 SNP markers that mapped to *R_13a_* (7 SNPs), *R_13b_* (3 SNPs), and *R_16_* (6 SNPs) were selected to test eight lines, including HA-R6 (*R_13a_*), RHA 397 (*R_13b_*), TX16R (*R_16_*), HA-R3 (*R_4_*), HA-R18 (*R_17_*), and HA-R19 (*R_18_*), and two lines, HA 89 and HA 434, as the respective susceptible parents in the *R_13a_* and *R_16_* mapping (Table 7). For the seven markers mapped to *R_13a_*, marker C13_194268343 could differentiate *R_13a_* from the remaining rust *R* genes, except for *R_13b_* (Figure 5a), while markers C13_195501970, C13_195522913, C13_195526945, and C13_195556768 could distinguish *R_13a_* from *R_13b_* and *R_17_*, but not the other *R* genes (Table 7a). All three markers mapped to *R_13b_*, S13_236323209, S13_236323867, and S13_237169906, could differentiate *R_13b_* from the rest of the *R* genes, except for *R_4_* (Table 7b, Figure 5b). Three of the six SNP markers mapped to *R_16_*, C13_194722668, C13_195605372, and C13_195874138, distinguished *R_16_* from the other five *R* genes (Table 7c; Figure 5c). These three SNP markers were further genotyped in the 96-line evaluation panel. Only SNP marker C13_194722668 could differentiate *R_16_* from the other 95 lines tested and is unique to *R_16_* (Figure 6a). The SNP, C13_195874138, could differentiate *R_16_* from the other 90 lines tested, but five lines, RNID, 803–1, RHA 417, RHA 295, and RHA 426, shared the *R_16_* marker allele with TX16R.

Three DM *R* genes, *Pl_17_*, *Pl_19_*, and *Pl_33_*, have been located in a gene cluster on chromosome 4 [32,34,40,76]. A total of 17 SNP markers used in the *Pl_33_* saturation mapping were selected to test four lines, TX16R (*Pl_33_*), HA 458 (*Pl_17_*), HA-DM5 (*Pl_19_*), and the susceptible parent, HA 434. Only SNP marker C4_5671004 can distinguish *Pl_33_* from *Pl_17_* and *Pl_19_*, while SNP marker C4_5562979 can distinguish *Pl_33_* from *Pl_19_* but not *Pl_17_*. Subsequently, these two markers, C4_5671004 and C4_5562979, were tested in a panel with 96 selected sunflower lines. As expected, the C4_5671004 marker allele was present only in the TX16R line, while the C4_5562979 marker allele was present in TX16R and in lines containing the *Pl_17_* gene (Figure 6b).

## 3. Discussion

Disease resistance genes tend to be clustered in the genome and are common across plants [77,78,79]. An *R* gene cluster with nine rust and eight DM *R* genes located on the lower end of sunflower chromosome 13 represents the largest *R*-gene cluster in sunflower. This *R* gene cluster can be further divided into two sub-clusters, sub-cluster I containing three rust *R* genes (*P_u6_*, *R_adv_*, and *R_11_*) and three fertility restorer genes (*Rf1*, *Rf5*, and *Rf7*) and sub-cluster II including six rust *R* genes (*R_4_*, *R_13a_*, *R_13b_*, and *R_16_*–*R_18_*) and eight DM *R* genes (*Pl_5_*, *Pl_8_*, *Pl_21_*, *Pl_22_*, *Pl_31_*, *Pl_32_*, *Pl_34_*, and *Pl_36_*) [36,37,38,40,42,64,73,80]. Six rust *R* genes (*R_4_*, *R_13a_*, *R_13b_*, and *R_16_*–*R_18_*) in sub-cluster II could be differentiated with race-specific resistance, except for the three, *R_13a_*, *R_13b_*, and *R_16_*, that exhibit resistance to all of the *P. helianthi* races that have been identified in North America thus far [73]. Polymorphic markers resulting from high-resolution mapping would be able to tackle this challenging region.

In previous studies, no marker could distinguish between *R_13a_* and *R_13b_* as these two genes are linked to a set of common markers [64,75]. Three genes, *R_13a_*, *R_13b_*, and *R_16_*, originated from different sources, with *R_13a_* from the plant introduction line PI 650,362 from France, *R_13b_* from an inbred line introduced from South Africa, and *R_16_* from the sunflower-wild *H. annuus* Texas-16 (Supplementary Table S2). Saturation mapping of *R_13a_*, *R_13b_*, and *R_16_* using a set of common sequencing-based SNP markers obtained in the current study revealed that four SNP markers, C13_195501970, C13_195526945, C13_195522913, and C13_195556768, could distinguish *R_13a_* from *R_13b_* and *R_16_*, while three SNP markers, S13_237169906, S13_236323867, and S13_236323209, mapped only to the *R_13b_* map (Figure 4a,b). Twelve SNP markers in the *R_16_* saturation map differentiated *R_16_* from *R_13a_* and *R_13b_* (Figure 4c). These results indicate that these three genes are different. Six SNP markers that were selected from whole-genome sequencing of HA-R6 (*R_13a_*) and TX16R (*R_16_*) were mapped distal to *R_13b_*; however, no new marker was mapped downstream of *R_13b_*. The lack of SNPs directly obtained from whole-genome sequencing of RHA 397 (*R_13b_*) may be a limitation in detecting more polymorphic markers in the HA-89/RHA 397 population.

Although the target regions of *R_13a_* and *R_16_* were saturated with the newly developed SNP markers, most markers were co-segregated with the genes in the saturation maps, especially for *R_13a_* (Figure 4). Fine mapping using whole-genome sequencing combined with large mapping populations was able to separate the co-segregated markers and place *R_13a_* and *R_16_* into a 790 kb region in the XRQr1.0 genome assembly. Molecular studies on disease *R* gene cloning have demonstrated that most *R* genes in crops encode nucleotide-binding leucine-rich repeat (NLR) motifs (for a review, see Wersch and Li 2019) [79]. The second largest NLR cluster has been reported on the lower end of chromosome 13, which corresponds to the two gene clusters in this region [81]. Four predicted NLR genes were found in the 790 kb target region of *R_13a_* and *R_16_* from the XRQr1.0 gene annotation (Table 6). The PacBio long read target region sequencing of *R_13a_* and *R_16_* and further functional analyses of the candidate genes can further help to reveal the molecular mechanism of rust resistance of the clustered genes.

Similar to *R_13a_* and *R_16_*, the DM *R* gene, *Pl_33_*, is also located in a gene cluster with five other DM *R* genes, *Pl_17_*, *Pl_19_*, *Pl_27_*, *Pl_28_*, and *Pl_29_*, at the upper end of sunflower chromosome 4 [32,34,38,40,76]. The differentiation of six DM *R* genes within this small region was achieved by whole-genome sequencing-based high-resolution mapping when traditional allelic analysis and resistance specificity to different pathotypes could not differentiate them. Based on the markers linked to genes, *Pl_27_* was mapped to a 5.4 Mb location on chromosome 4, while *Pl_28_* and *Pl_29_* were located in a region between nucleotide positions 6.62 and 7.01 Mb, respectively, on the XRQr1.0 assembly, close to *Pl_19_* [38,76]. Our recent fine mapping of *Pl_17_*, *Pl_19_*, and *Pl_33_* revealed that *Pl_33_* is close to *Pl_17_* in a region between nucleotide positions of 5.69–5.71 Mb on the XRQr1.0 assembly, while *Pl_19_* is located 1 Mb from *Pl_17_* and *Pl_33_* (Table 5). Meanwhile, the diagnostic markers developed for *Pl_17_*, *Pl_19_*, and *Pl_33_* could clearly distinguish them. A disease defense-related NLR gene, HanXRQChr04g0095641, was found in the target region on the XRQr1.0 genome assembly as a candidate gene for both *Pl_17_* and *Pl_33_*. Large-scale sequence analyses of complex *R* gene haplotypes will shed light on the processes of diversifying resistance specificities in the cluster in the future.

Resistance against DM and rust is controlled by single dominant genes in sunflower. Resistance genes could be ineffective during coevolution with pathogens in which some pathogens can quickly change their genomic components by mutation or recombination when selective pressure is favored [82]. *R* gene pyramiding is a commonly accepted, effective method to create durable resistance in crops [83,84]. It is more feasible to combine *R_13a_*, *R_13b_*, *R_16_*, and *Pl_33_* with disease resistance genes from other chromosomes or from the same chromosomes but at distal locations due to the increased possibility of linkage. Combining *R* genes within similar regions is still achievable and, in some instances, induced recombination is required by utilizing a large population to screen for few recombinants. To achieve this, diagnostic molecular markers for each gene are prerequisites. In the present study, the map resolution for each of the genes studied was greatly increased, and the tightly linked diagnostic markers for *R_13a_*, *R_13b_*, *R_16_*, and *Pl_33_* would be important practical implications for tracking gene introgression to elite sunflower lines and pyramiding these genes to slow pathogen evolution to evade *R*-gene and enhance *R*-gene durability.

## 4. Materials and Methods

### 4.1. Mapping Populations and Evaluation Panel

The F_2_ populations for *R_13a_* and *R_13b_* saturation mapping of additional markers in the present study were initially created from crosses between HA 89 and HA-R6 (carrying *R_13a_*)/RHA 397 (carrying *R_13b_*), respectively, with 140 individuals each, which were previously used to map *R_13a_* and *R_13b_* to sunflower chromosome 13 [64,75]. HA 89 is an oilseed maintainer line used as a susceptible parent. Both HA-R6 (PI 607509) and RHA 397 (PI 597374) are resistant to rust, and HA-R6 is a confection sunflower line, while RHA 397 is a male fertility restorer line of oilseed sunflower [64]. For the fine mapping, recombinants were screened from 2820 F_3_ individuals selected from the previously characterized F_2:3_ families that were heterozygous for *R_13a_*. Each selected heterozygous F_3_ family equates to a segregated F_2_ population.

Saturation mapping of the rust *R* gene *R_16_* and DM *R* gene *Pl_33_* was performed in the F_2_ population developed from the cross of HA 434 and TX16R (carrying *R_16_* and *Pl_33_*) with 146 and 148 F_2_ individuals, respectively, which were previously used for the initial mapping of *R_16_* and *Pl_33_* to sunflower chromosomes 13 and 4, respectively [40]. HA 434 (PI 633744) is an oilseed line susceptible to DM and rust, while TX16R (PI 642072) is resistant to sunflower DM, rust, and SuMV [40]. For the fine mapping, recombinants were screened from 2256 F_3_ individuals selected from the previously characterized F_2:3_ families that were heterozygous for both *R_16_* and *Pl_33_*, which was equal to a segregated F_2_ population for both genes.

The specificity of the DNA markers for *R_13a_*, *R_16_*, and *Pl_33_* was evaluated among 96 sunflower inbred lines with diverse origins, including 24 and 17 lines harboring different DM and rust *R* genes, respectively (Supplementary Table S3).

### 4.2. Whole-Genome Sequencing and SNP/Indel Calling

Sunflower lines HA-R6 (*R_13a_*) and TX16R (*R_16_* and *Pl_33_*) were sequenced separately at the whole-genome level with 40× genome coverage on the Illumina HiSeq sequencing platform by Novogene Inc. according to their protocols. The genomic DNA of each sample was randomly sheared into short fragments of about 350 bp, respectively. The obtained fragments were subjected to library construction using the NEBNext^®^ DNA Library Prep Kit, with strictly following the instructions. Briefly, as followed by end repairing, dA-tailing, and further ligation with NEBNext adapter, the required fragments (in 300–500 bp size) were PCR enriched by P5 and indexed P7 oligos. After purification and subsequent quality check, pair-end sequencing was performed on Illumina^®^ sequencing platform, with the read length of PE150 bp at each end. The raw reads containing adaptors, reads with >1% ambiguous bases, and reads with low quality (greater than 50% bases less than 15 Q score) were removed and excluded from further analysis. For HA-R6, totally 141.9 G raw data were sequenced from this run, with 141.8 G clean data generated after filtering low-quality data. For TX16R, totally 178.3 G raw data were sequenced from this run, with 178.2 G clean data generated after filtering low-quality data. The clean reads were aligned to the two reference genomes of XRQr1.0 (https://www.heliagene.org/HanXRQ-SUNRISE/ (accessed on 10 April 2019)) and HA412-HO (https://www.heliagene.org/HA412.v1.1.bronze.20141015/ (accessed on 10 April 2019)), respectively. All SNPs and InDels were identified by using the mapped reads. The SNPs in the targeted gene regions were selected based on their physical positions along chromosomes 4 or 13, and the flanking sequences of each SNP were extracted from the XRQr1.0 and HA412-HO reference assemblies (Supplementary Table S4).

### 4.3. SNP Marker Selection from Whole-Genome Sequencing

Both *R_13a_* and *R_16_* were previously mapped to a similar region located at the lower end of sunflower chromosome 13. A total of 308 SNPs were selected based on SNPs/InDels between HA-R6 carrying *R_13a_* and the two reference genomes in the target region of chromosome 13 with 116 selected from the HA412-HO genome and 192 selected from the XRQr1.0 genome. Another set of 124 SNPs was selected based on SNPs/InDels between TX16R (*R_16_*) and the XRQr1.0 reference in a similar region of chromosome 13. The HA412-HO whole-genome sequence was assembled from Illumina reads (100 bp) and 454 Roche reads (400*–*1000 bp) of the inbred line HA412-HO, while the XRQ whole-genome sequence was assembled from PacBio sequencing data with an average read length of 10.3 kb of the inbred line XRQ. The two sunflower reference sequences provide alternative opportunities for SNP discovery. The SNP markers were named with the prefixes C13 or S13 followed by a number representing the physical positions of the SNPs along chromosome 13 of each reference genome assembly (Supplementary Table S4). C13 represent the SNPs from the XRQr1.0 reference genome, while prefixes S13 represent the SNPs from the HA412-HO reference genome.

*Pl_33_* was previously mapped to a similar position as *Pl_17_* on chromosome 4 [40]. Thirty-two SNPs from the variants between the TX16R (*Pl_33_*) whole genome sequence and the XRQr1.0 reference genome sequence located in the target region of chromosome 4 were selected for marker development. An additional 125 SNPs that were selected from our *Pl_17_* fine mapping project were also used for marker development in the current study [76]. The SNP markers were named with the prefixes C4 or S4 followed by a number representing the physical positions of the SNPs along chromosome 4 of each reference genome assembly. C4 represents the SNPs from the XRQr1.0 reference genome, while prefixes S4 represents the SNPs from the HA412-HO reference genome (Supplementary Table S4).

Other SSR and SNP markers associated with three target genes from previous studies is listed in Supplementary Table S5.

### 4.4. PCR-Based Genotyping of SNP Markers and Linkage Analysis

PCR-based length polymorphic SNP primers were designed by using the Primer 3-based Primer-BLAST suite embedded within the NCBI website (https://www.ncbi.nlm.nih.gov/tools/primer-blast/ (accessed on 16 August 2019)). The artificial mismatches and length polymorphisms for the SNP primers were created (Supplementary Table S6) as described by Qi et al. (2016) [33] and Long et al. (2017) [85] based on SNP flanking sequences. Polymerase chain reaction (PCR) for SNPs was conducted as described by Ma et al. (2020) [86], and the amplicons were separately visualized and scored on 6.5% polyacrylamide gel using an IR2 4300/4200 DNA analyzer (LI-COR, Lincoln, NE, USA).

After scoring each marker, the genotype data were chi-square (χ^2^) tested for goodness-of-fit to evaluate whether the segregation ratio for each marker fit the Mendelian ratios, e.g., 1:3 for dominant and 1:2:1 for codominant. Markers fitting the Mendelian ratios were used for linkage analysis with either the respective rust or DM phenotype data by using JoinMap 4.1 software, in which a regression mapping algorithm and Kosambi’s mapping function were selected [87]. The cutoffs for the linkage analysis among markers were set at a likelihood of odds (LOD) ≥ 3.0 and maximum genetic distance ≤ 50 centimorgans (cM).

### 4.5. Rust Evaluation of Recombinants

The *R_13a_* and *R_16_* recombinants, together with their respective parents, HA 89 and HA-R6 for *R_13a,_* and HA 434 and TX16R for *R_16_*, were evaluated for their reactions to rust infection following the method of Qi et al. (2011) [57]. Plants at the four-leaf stage were inoculated with *P. helianthi* race 336, and the disease reactions were scored for their infection types (ITs) based on a 0–4 scale and the percentage of leaf area covered with pustules (severity) at 12–14 days after inoculation [88,89]. Infection types 0, 1, and 2, when combined with a pustule coverage of 0–0.5%, were classified as resistant, and ITs 3 and 4 with pustule coverages greater than 0.5%, were considered to be susceptible.

### 4.6. Downy Mildew Evaluation of Recombinants

The *Pl_33_* recombinants selected from the segregated population using its flanking markers, together with two parents, HA 434 and TX16R, were tested for DM resistance with an isolate of *P. halstedii* race 734 by using the whole seedling immersion method, as described by Gulya et al. (1999) [90] and Qi et al. (2015) [32]. Briefly, approximately 40 seeds from each recombinant family were germinated, and at least 30 seedlings from each recombinant family were inoculated with *P. halstedii* race 734 after 2–3 days. After sporulation, the seedlings were evaluated for disease resistance and susceptibility, in which susceptible seedlings showed sporulation on their cotyledons and true leaves, and resistant seedlings showed no sporulation. The genotype of each recombinant was determined as homozygous susceptible if all seedlings in the recombinant family showed sporulation on the cotyledons and true leaves, homozygous resistant if none of the seedlings exhibited sporulation, and segregated if some seedlings showed sporulation on the cotyledons and true leaves while some showed no sporulation.

## Figures and Tables

**Table 1:** Genetic and physical positions of the SNP markers linked to *R_13a_* on a fine map of sunflower chromosome 13.

Marker	No. Recombination	Position in the Fine Map (cM)	Physical Position on XRQr1.0 Assembly (bp)	Physical Position on HA412-HO Assembly (bp)
SFW01497	0	0	193,089,467–193,089,349	236,437,096–236,436,978
C13_194268343	30	0.53	194,268,143–194,268,543	-
C13_194735854	5	0.62	194,735,654–194,736,054	235,097,621–235,097,389
C13_194757055	4	0.69	194,756,855–194,757,255	236,982,689–236,982,486
*R_13a_*	7	0.82	-	-
C13_195501970	20	1.17	195,501,770–195,502,170	-
C13_195522913	0	1.17	195,522,713–195,523,113	-
C13_195526945	0	1.17	195,526,745–195,527,145	-
C13_195556768	0	1.17	195,556,568–195,556,968	-
SFW04275	21	1.54	196,464,687–196,464,768	238,083,828–238,083,909
SFW04317	1	1.56	196,474,077–196,473,983	238,092,624–238,092,530
SFW05743	4	1.63	196,521,145–196,521,026	238,196,827–238,196,708
HT382	106	3.51	-	-

**Table 2:** Genetic and physical positions of the SNP markers linked to *R_16_* on a fine map of sunflower chromosome 13.

Marker	No. Recombination	Position in the Fine Map (cM)	Physical Position on XRQr1.0 Assembly (bp)	Physical Position on HA412-HO Assembly (bp)
ORS316	0	0	-	232,376,160 *
SFW8875	110	2.44	193,131,235–193,131,123	236,146,953–236,147,065
C13_194722668	43	3.39	194,722,468–194,722,868	
*R_16_*	8	3.57	-	-
C13_195512786	6	3.70	195,512,586–195,512,986	-
C13_195552917	0	3.70	195,552,717–195,553,117	-
C13_195605372	0	3.70	195,605,172–195,605,572	-
C13_195836770	7	3.86	195,836,570–195,836,970	-
C13_195840634	0	3.86	195,840,434–195,840,834	-
C13_195874138	0	3.86	195,873,938–195,874,338	-
SFW05743	31	4.54	196,521,145–196,521,026	238,196,827–238,196,708

* reverse primer aligns to HA412-HO sequences.

**Table 3:** Genetic and physical positions of the SNP markers linked to *Pl_33_* on a fine map of sunflower chromosome 4.

Marker	No. Recombination	Position in the Fine Map (cM)	Physical Position on XRQr1.0 Assembly (bp)	Physical Position on HA412-HO Assembly (bp)
SFW04052	0	0	4,208,180–4,208,271	3,621,090–3,621,181
C4_5261411	7	0.16	5,261,211–5,261,611	-
C4_5562979	11	0.40	5,562,779–5,563,179	6,160,956–6,160,556
C4_5641353	1	0.42	5,641,153–5,641,553	13,535,870–13,536,270
C4_5671004	0	0.42	5,669,804–5,671,204	6,082,367–6,082,024
*Pl_33_*	0	0.42	-	-
SBP006	0	0.42	5,703,949–5,704,083	5,947,481–5,947,612
SBP005	1	0.44	5,704,420–5,704,545	5,947,019–5,947,144
C4_5704814	0	0.44	5,704,614–5,705,014	6,029,066–6,028,666
C4_5738736	0	0.44	5,738,536–5,738,936	5,993,332–5,992,932
SFW06856	71	2.02	6,978,325–6,978,206	7,872,910–7,873,029

**Table 4:** Map positions of the SNP markers linked to *R_13a_*, *R_13b_*, and *R_16_* on sunflower chromosome 13.

SNP Marker	*R_13a_* Saturation Map (cM)	*R_13a_* Fine Map (cM)	*R_13b_* Saturation Map (cM)	*R_16_* Saturation Map (cM)	*R_16_* Fine Map (cM)	Physical Position on XRQr1.0 Assembly (bp)	Physical Position on HA412-HO Assembly (bp)
S13_236323867	-	-	0.79	-	-	-	236,323,667–236,324,067
S13_236323209	-	-	1.01	-	-	-	236,323,009–236,323,409
SFW01497	0.00	0.00	1.01	-	-	193,089,467–193,089,349	236,437,096–236,436,978
S13_237169906	-	-	1.01	-	-	-	237,169,706–237,170,106
C13_194268343	0.00	0.53	1.01	5.03	NT	194,268,143–194,268,543	-
C13_194722668	-	-	-	6.75	3.39	194,722,468–194,722,868	236,711,421–236,711,022
C13_194735854	0.00	0.62	1.01	-	-	194,735,654–194,736,054	235,097,621–235,097,389
C13_194757055	0.00	0.69	1.01	-	-	194,756,855–194,757,255	236,982,689–236,982,486
*R_13b_*	-	-	1.44	-	-	-	-
*R_13a_*	0.00	0.82	-	-	-	-	-
C13_195501970	0.00	1.17	-	-	-	195,501,770–195,502,170	-
*R_16_*	-	-	-	6.75	3.57	-	-
C13_195512786		-	-	6.75	3.70	195,512,586–195,512,986	-
C13_195522913	0.00	1.17	-	-	-	195,522,713–195,523,113	-
C13_195526945	0.00	1.17	-	-	-	195,526,745–195,527,145	-
C13_195552917	-	-	-	6.75	3.70	195,552,717–195,553,117	-
C13_195556768	0.00	1.17	-	-	-	195,556,568–195,556,968	-
C13_195605372	-	-	-	6.75	3.70	195,605,172–195,605,572	-
C13_195836770	-	-	-	7.11	3.86	195,836,570–195,836,970	-
C13_195840634	-	-	-	7.11	3.86	195,840,434–195,840,834	-
C13_195874138	-	-	-	7.11	3.86	195,873,938–195,874,338	-
SFW04275	0.00	1.54	4.06	-	-	196,464,687–196,464,768	238,083,828–238,083,909
SFW04317	0.00	1.56	4.06	7.83	NT	196,474,077–196,473,983	238,092,624–238,092,530
SFW05743	0.70	1.63	4.06	8.17	4.54	196,521,145–196,521,026	238,196,827–238,196,708

NT: Not test in fine mapping.

**Table 5:** Map positions of the SNP markers linked to *Pl_17_*, *Pl_19_*, and *Pl_33_* on sunflower chromosome 4.

SNP Marker	*Pl_17_* Fine Map (cM) ^†^	*Pl_19_* Fine Map (cM) ^†^	*Pl_33_* Fine Map (cM)	Physical Position on XRQr1.0 Assembly (bp)	Physical Position on HA412-HO Assembly (bp)
C4_5261411	-	-	0.1551	5,261,211–5,261,611	-
C4_5562979	-	-	0.3989	5,562,779–5,563,179	6,160,956–6,160,556
C4_5641353	-	-	0.4212	5,641,153–5,641,553	13,535,870–13,536,270
C4_5671004 *	-	-	0.4212	5,669,804–5,671,204	6,082,367–6,082,024
*Pl_33_*	-	-	0.4212	-	-
C4_5696413 **	0.26595	-	-		
C4_5704814	-	-	0.4435	5,704,614–5,705,014	6,029,066–6,028,666
C4_5705018 **	0.26595	-	-	5,704,818–5,705,218	6,028,462–6,028,862
C4_5705841 **	0.28257	-	-	5,705,641–5,706,041	6,027,639–6,028,039
C4_5709499 **	0.28257	-	-	5,709,299–5,709,699	6,021,349–6,021,749
C4_5711524	0.28257	-	-	5,711,324–5,711,724	6,627,884–6,628,284
*Pl_17_*	0.31581	-	-	-	-
SPB0001 **	0.34905	-	-	5,696,076–5,696,181	5,950,918–5,951,024
SPB0006	0.34905	-	0.4212	5,703,949–5,704,083	5,947,481–5,947,612
SPB0005	0.34905	-	0.4435	5,704,420–5,704,545	5,947,019–5,947,144
C4_5738736	-	-	0.4435	5,738,536–5,738,936	5,993,332–5,992,932
C4_6675662 ***	-	0.4212	-	6,675,462–6,675,862	6,972,167–6,972,567
C4_6676629 ***	-	0.4212	-	6,676,429–6,676,829	6,971,201–6,971,601
*Pl_19_*	-	0.4655	-	-	-
C4_6711381	-	0.6428	-	6,711,181–6,711,581	7,089,348–7,089,748
C4_6730143	-	0.7536	-	6,729,943–6,730,343	7,073,422–7,073,822
S4_7964876 ***	-	1.1304	-	6,914,409–6,914,809	7,964,676–7,965,076

^†^ map data for *Pl_17_* and *Pl_19_* were taken from Ma et al., 2019 [76]. * diagnostic SNP markers specific to *Pl_33_*, ** diagnostic SNP markers specific to *Pl_17_*, and *** diagnostic SNP markers specific to *Pl_19_*.

**Table 6:** Predicted plant disease defense-related genes in the interval of *R_13a_*, *R_16_*, and *Pl_33_* in the XRQr1.0 genome assembly.

Candidate Gene	Description	Physical Position (bp)	Length (bp)
***R_13a_* and *R_16_* interval**		194,722,468–195,512,986	790,518
HanXRQChr13g0425851	Putative NB-ARC; P-loop containing nucleoside triphosphate hydrolase; Leucine-rich repeat domain, L domain-like	194,725,998–194,753,531	27,534
HanXRQChr13g0425891	Putative NB-ARC; P-loop containing nucleoside triphosphate hydrolase; Leucine-rich repeat domain, L domain-like	194,800,201–194,803,684	3484
HanXRQChr13g0425931	Putative NB-ARC; P-loop containing nucleoside triphosphate hydrolase; Leucine-rich repeat domain, L domain-like	195,196,820–195,210,745	13,926
HanXRQChr13g0425941	Putative NB-ARC; P-loop containing nucleoside triphosphate hydrolase; Leucine-rich repeat domain, L domain-like	195,250,038–195,252,703	2666
***Pl_33_* interval**		5,641,153–5,704,545	63,392
HanXRQChr04g0095641	Probable disease resistance protein (TIR-NBS-LRR class) family	5,672,715–5,705,044	32,330

**Table 7:** (**a**) Specificity test of SNP markers linked to *R_13a_* in eight lines, (**b**) Specificity test of SNP markers linked to *R_13b_* in eight lines, (**c**) Specificity test of SNP markers linked to *R_16_* in eight lines.

(**a**)								
**Marker**	**HA 89**	**HA 434**	**HA-R6/*R_13a_***	**RHA 397/*R_13b_***	**TX16R/*R_16_***	**HA-R3/*R_4_***	**HA-R18/*R_17_***	**HA-R19/*R_18_***
C13_194268343	A	A	B	B	C	H	H	H
C13_194735854	A	A	B	B	B	B	H	B
C13_194757055	A	A	B	B	B	B	H	B
C13_195501970	A	A	B	A	B	B	A	B
C13_195522913	A	A	B	A	B	B	A	B
C13_195526945	A	A	B	A	B	B	A	B
C13_195556768	A	A	B	A	B	B	A	B
A represents HA 89 marker allele; B represent HA-R6 marker allele; C represents the marker allele different A and B, H represents heterozygous.								
(**b**)								
**Marker**	**HA 89**	**HA 434**	**HA-R6/*R_13a_***	**RHA 397/*R_13b_***	**TX16R/*R_16_***	**HA-R3/*R_4_***	**HA-R18/*R_17_***	**HA-R19/*R_18_***
S13_236323209	A	A	A	B	A	B	A	C
S13_236323867	A	A	A	B	A	B	A	C
S13_237169906	A	A	A	B	A	B	A	C
A represents HA 89 marker allele; B represent RHA 397 marker allele; C represents the marker allele different A and B.								
(**c**)								
**Marker**	**HA 89**	**HA 434**	**HA-R6/*R_13a_***	**RHA 397/*R_13b_***	**TX16R/*R_16_***	**HA-R3/*R_4_***	**HA-R18/*R_17_***	**HA-R19/*R_18_***
C13_194722668	A	A	A	A	B	A	A	A
C13_195552917	A	A	B	A	B	C	A	B
C13_195605372	A	A	A	A	B	A	A	A
C13_195836770	A	A	B	B	B	B	A	B
C13_195840634	A	A	B	B	B	B	A	B
C13_195874138	A	A	A	A	B	A	A	A
A represents HA 89 marker allele; B represent TX16R marker allele; C represents the marker allele different A and B.								

## Supplementary Materials

The following are available online at https://www.mdpi.com/article/10.3390/ijms23179516/s1.
